# Antenatal ultrasound diagnosis of fetal micrognathia: validation and reproducibility of quantitative methods

**DOI:** 10.1002/uog.70137

**Published:** 2025-11-20

**Authors:** M. C. Denk, M. Bertin, V. Donadono, L. Kangesu, A. Yulia, F. Ushakov, P. P. Pandya, R. Napolitano

**Affiliations:** ^1^ Fetal Medicine Unit University College London Hospitals NHS Foundation Trust London UK; ^2^ North Thames Cleft Service Great Ormond Street Hospital London UK; ^3^ St Andrews Centre Broomfield Hospital Chelmsford UK; ^4^ Elizabeth Garrett Anderson Institute for Women's Health University College London London UK

**Keywords:** fetal ultrasound, mandible angle measurements, micrognathia, pregnancy, prenatal diagnosis, reproducibility

## Abstract

**Objectives:**

The antenatal ultrasound diagnosis of fetal micrognathia can be made subjectively or objectively. We aimed to validate quantitative ultrasound methods proposed for the diagnosis of fetal micrognathia in the second and third trimesters of pregnancy by evaluating their diagnostic performance, reproducibility and impact of measurement variability on diagnosis.

**Methods:**

This was a retrospective study analyzing the objective diagnostic accuracy, reproducibility and impact of measurement variability of mandible angle measurements from facial profile images of fetuses with suspected micrognathia diagnosed antenatally using a subjective method. Fetuses were recruited at between 18 and 28 weeks' gestation, at University College London Hospital, UK, between 2000 and 2018. Four quantitative methods were compared: measurement of the inferior facial angle (IFA), the fronto–naso–mental angle (FNMA), the maxilla–nasion–mandible angle (MNMA) and the facial maxillary angle (FMA). Methods were validated by calculating the percentage of cases in which the measured angles met the diagnostic criteria for micrognathia. To assess intra‐ and interobserver reproducibility, two sonographers, blinded to each other's findings, measured each angle twice. Bland–Altman plots were used to calculate mean systematic differences and 95% limits of agreement (LoA). Data were expressed in degrees and as percentages to account for the increase in fetal size with advancing gestation. The impact of measurement variability was evaluated by recalculating the diagnostic accuracy adding to the angle cut‐off the intraobserver 95% LoA.

**Results:**

Of 132 fetuses diagnosed with suspected antenatal micrognathia, 84 cases had a known outcome (termination of pregnancy (*n* = 56); fetal demise (*n* = 11); neonatal death (*n* = 4); live birth (*n* = 13)) and 48 were lost to follow‐up. Fetal facial profile ultrasound images were available for 49 cases in which reproducibility of the measurements was assessed (termination of pregnancy (*n* = 22); fetal demise (*n* = 5); neonatal death (*n* = 3); live birth (*n* = 6); lost to follow‐up (*n* = 13)). FNMA was the most reproducible and FMA was the least reproducible measurement. Validation analysis showed that 14%, 100%, 88% and 90% of fetuses from our cohort met the criteria for the diagnosis of micrognathia using the cut‐off criteria suggested in the original studies for IFA, FNMA, MNMA and FMA, respectively. The 95% LoA for intra‐ and interobserver reproducibility were 3.5% and 9.5% for FNMA, and 11.6% and 45.0% for FMA, respectively. When adding the intraobserver widest random error to the original study cut‐offs, the percentage of fetuses meeting the criteria for diagnosis decreased to 0%, 87%, 49% and 45% for IFA, FNMA, MNMA and FMA, respectively.

**Conclusions:**

Current quantitative ultrasound methods to assess micrognathia antenatally have variable diagnostic accuracy and are not consistently reproducible, which can lead to overdiagnosis or missed diagnosis. Measurement of FNMA is the preferred method to support subjective assessment, however, further evaluation in prospective studies is warranted. © 2025 The Author(s). *Ultrasound in Obstetrics & Gynecology* published by John Wiley & Sons Ltd on behalf of International Society of Ultrasound in Obstetrics and Gynecology.

## INTRODUCTION

The fetal mandible is a common site for defects induced by a large number of genetic conditions and adverse environmental factors. The complex development of the fetal mandible requires embryonic components to interact and fuse, and any defects in this multistep process may be difficult to visualize antenatally on ultrasound^1^.

Normal fetal mandible development begins from 7 weeks' gestation and is complete at 16 weeks, but defects may be visible later in gestation or after birth^2^. Micrognathia is a rare facial malformation characterized by a small, underdeveloped mandible. It is associated frequently with retrognathia, in which the mandible is receded^3^.

Prenatal diagnosis of micrognathia is important, since it may be associated with adverse neonatal outcomes such as acute neonatal respiratory distress syndrome, which potentially require interventions at birth^4^. The prenatal diagnosis of micrognathia can be confirmed in second‐ and third‐trimester fetuses by the sonographic detection of a small chin in the sagittal view of the fetal facial profile. However, acquisition of a midline facial profile is not recommended at routine fetal anomaly screening in the UK^5^, ^6^. International guidelines are inconsistent, recommending the fetal facial profile image as an optional or as an essential plane of acquisition at either a basic or a detailed anomaly screening^7^, ^8^. Furthermore, there is no consensus on what constitutes a normal fetal facial profile other than on subjective assessment. Objective quantitative techniques for the ultrasound diagnosis of fetal micrognathia have been proposed but there is no agreement on which one should be used. Most evaluate the midsagittal view of the facial profile and assess the angle between the mandible and a second identifiable structure of the fetal profile image from 18 weeks to a later gestation. It has been reported that the sensitivity for the antenatal diagnosis of micrognathia is between 55% and 100%^9^. Furthermore, subjective *vs* objective methods show discrepancies of between 55% and 72–100%^10^, ^11^.

To the best of our knowledge, the reproducibility of different methods for the ultrasound diagnosis of micrognathia has not been evaluated in the same cohort of fetuses. The aims of this study were to validate quantitative methods for the antenatal diagnosis of micrognathia on ultrasound in the second and third trimesters of pregnancy and to evaluate their reproducibility and impact of measurement variability on the rate of diagnosis.

## METHODS

This was a retrospective study of data collected between 2000 and 2018, including fetuses diagnosed antenatally with suspected micrognathia on subjective ultrasound assessment at between 18 and 28 weeks' gestation by a fetal medicine specialist at University College London Hospital, London, UK. Micrognathia can be diagnosed before or after this gestational‐age range, however, the lower limit of gestational age was chosen as 18 weeks because this is the time when midtrimester fetal anatomy screening is recommended in the UK. Following expert consensus (M.C.D., M.B., R.N.), the upper limit was chosen as 28 weeks, when fetal facial profile abnormalities may still be observed as a coincidental finding during a fetal growth scan or in case of late anomaly screening in pregnancies presenting late for antenatal care.

**Figure 1:**
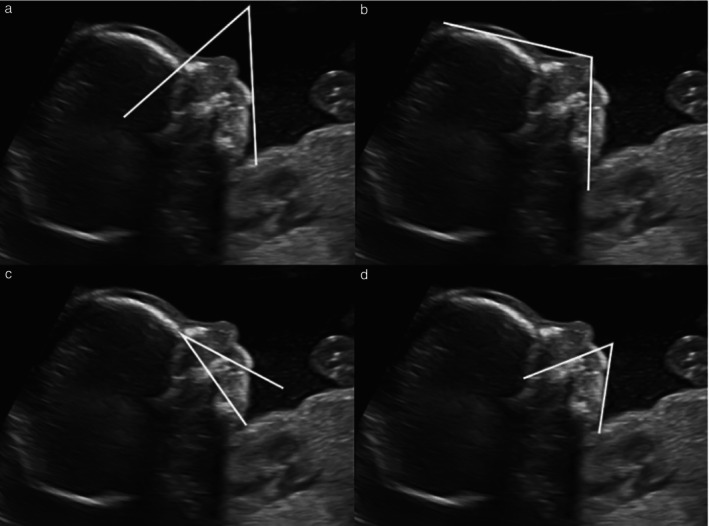
Two‐dimensional grayscale ultrasound images showing quantitative angle measurements on the facial profile of a fetus at 22 weeks' gestation diagnosed antenatally with suspected micrognathia. (a) Inferior facial angle: angle between line perpendicular to forehead and line joining mentum and protrusive lip. Diagnostic threshold for micrognathia, < 50°. (b) Fronto–naso–mental angle: angle between line from most prominent point of bony part of fetal forehead (os frontale) to tip of visible soft tissue of nose and line from most prominent point of soft tissue of fetal chin (mandible) to tip of visible soft tissue of nose. Diagnostic threshold for micrognathia, < 142°. (c) Maxilla–nasion–mandible angle: angle between intersection of maxilla–nasion and mandible–nasion lines. Diagnostic threshold for micrognathia, > 17°. (d) Facial maxillary angle: angle between line overlying maxilla and line across mentum tip and upper lip. Diagnostic threshold for micrognathia, < 66°.

After an extensive literature review of different ultrasound methods proposed for the diagnosis of fetal micrognathia, expert consensus (M.C.D., M.B., R.N.) and feasibility of measurements, we selected four quantitative methods that involved angle measurement on still fetal facial profile ultrasound images, stored from cases with antenatally suspected micrognathia. The angles measured were: the inferior facial angle (IFA)^12^, the fronto–naso–mental angle (FNMA)^13^, the maxilla–nasion–mandible angle (MNMA)^14^ and the facial maxillary angle (FMA)^15^ (Figure 1). Two sonographers (M.C.D., M.B.), blinded to each other's caliper placement, after a standardization session, independently measured each angle twice using an offline software facility that is part of the obstetric image database (Viewpoint version 5; GE Healthcare, Munich, Germany). The sonographers had achieved competence in obstetric ultrasound and fetal medicine after completing a dedicated master programme^16^. Validation was performed to explore the diagnostic accuracy of each angle using the first measurement obtained by the first observer at the time of the reproducibility study. Intra‐ and interobserver reproducibility were assessed for each method using Bland–Altman plots^17^.

Data are reported in degrees and as percentages to account for the increase in fetal size with advancing gestation in line with previous methodology^16^, ^17^, ^18^, ^19^, ^20^. A single limit of agreement (LoA) was calculated using the average of the upper and lower limits.

A power calculation was performed to obtain adequate sample size according to previous reports^16^, ^17^, ^18^, ^19^, ^20^, and we estimated that a total of 42 measurements would be needed to detect a 30% difference in 95% LoA between two methods (31 measurements per method), with 80% power (alpha, 0.05). A sample of more than 45 images was considered adequate to achieve this.

Intra‐ and interobserver reproducibility were compared using paired Student's *t*‐test to assess the difference between 95% LoA percentages of repeated measurements^18^; differences were considered significant if *P* was < 0.05.

A single cut‐off for the diagnosis of micrognathia was chosen for each angle, selected based on previous original studies (< 50° for IFA, < 142° for FNMA, > 17° for MNMA and < 66° for FMA)^21^. In order to explore the impact of reproducibility on the antenatal diagnosis of micrognathia, the percentage of fetuses meeting the diagnostic criteria was calculated using the proposed above cut‐offs ± the 95% random error (i.e. intraobserver LoA) using Microsoft Excel.

Individual patient consent and ethical committee approval were not obtained, as images and data were analyzed anonymously without affecting pregnancy care. We interrogated the UK Health Research Authority tool (www.hra‐decisiontools.org.uk/ethics), which confirmed that ethical approval was not required.

## RESULTS

We identified 132 singleton pregnancies with a fetus diagnosed antenatally with suspected micrognathia at between 18 and 28 weeks' gestation. Among these 132 fetuses, 84 cases had a known outcome and confirmation of micrognathia after birth (termination of pregnancy (*n* = 56); fetal demise (*n* = 11); neonatal death (*n* = 4); live birth (*n* = 13)). In total, 48 fetuses were lost to follow‐up.

**Figure 2:**
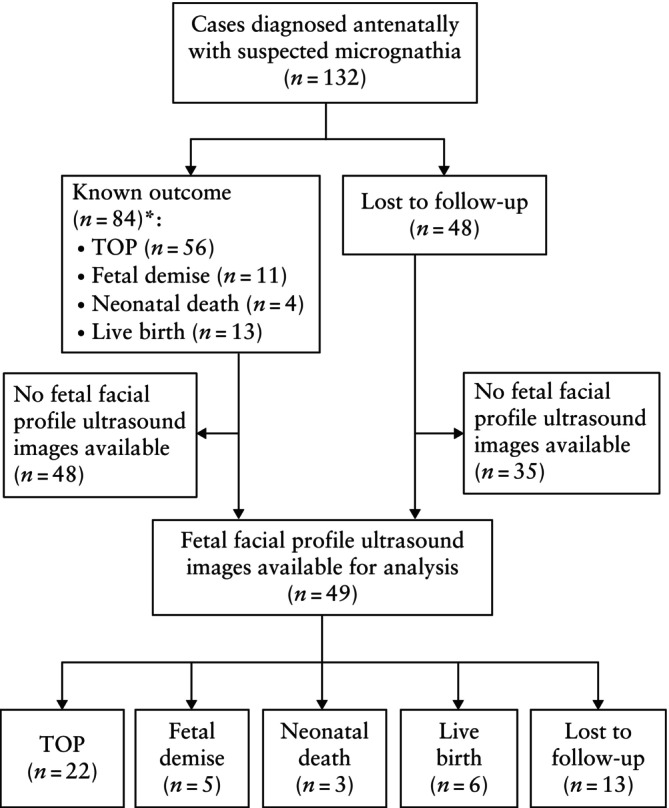
Flowchart summarizing inclusion of facial profile ultrasound images from fetuses diagnosed antenatally with suspected micrognathia, in which reproducibility of quantitative angle measurements was assessed. *And confirmation of micrognathia after birth. TOP, termination of pregnancy.

Of the 132 fetuses, a facial profile ultrasound image was available for 49 cases (termination of pregnancy (*n* = 22); fetal demise (*n* = 5); neonatal death (*n* = 3); live birth (*n* = 6); lost to follow‐up (*n* = 13)) in which reproducibility of the measurements was assessed (Figure 2).

**Figure 3:**
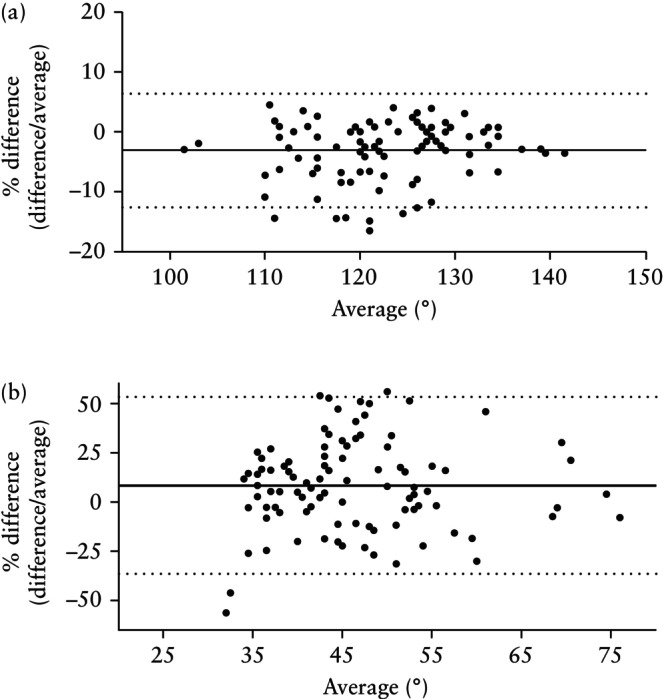
Bland–Altman plots of interobserver reproducibility of quantitative measurement of fetal profile angles on ultrasound for antenatal diagnosis of micrognathia. Most (a) (fronto–naso–mental angle, 9.5%) and least (b) (facial maxillary angle, 45.0%) reproducible angle measurements are shown. Solid lines are mean percentage difference and dotted lines are 95% limits of agreement.

The validation analysis revealed that 14%, 100%, 88% and 90% of fetuses from our cohort met the criteria for the diagnosis of micrognathia using the suggested cut‐offs in the original studies for IFA, FNMA, MNMA and FMA, respectively. The overall intraobserver reproducibility was < 6° (< 15%) and the overall interobserver reproducibility was < 21° (< 45%). FNMA was the most reproducible and FMA was the least reproducible measurement. The 95% LoA for intra‐ and interobserver reproducibility were 4.2° (3.5%) and 11.4° (9.5%) for FNMA and 5.2° (11.6%) and 20.9° (45.0%) for FMA, respectively (Figures 3 and S1, Table 1). Intraobserver reproducibility was better than interobserver reproducibility for all angle measurements (*P* < 0.05 for all). After one‐to‐one interobserver reproducibility comparisons, FNMA was more reproducible than any of the other three methods (*P* < 0.05); there was no difference between IFA and MNMA, and IFA was significantly more reproducible than FMA (*P* < 0.05) (Table S1).

**Table 1:** Reproducibility of quantitative fetal facial profile angle measurements for antenatal diagnosis of micrognathia on ultrasound

Parameter	Mean difference ( ° (%))	95% LoA ( ° (%))
IFA		
Intraobserver	0.5 (0.8)	5.3 (9.8)
Interobserver	9.0 (15.5)	20.4 (34.8)
FNMA		
Intraobserver	−0.1 (−0.1)	4.2 (3.5)
Interobserver	−3.8 (−3.2)	11.4 (9.5)
MNMA		
Intraobserver	0.0 (0.1)	3.0 (14.6)
Interobserver	2.9 (13.2)	6.3 (30.1)
FMA		
Intraobserver	0.4 (0.6)	5.2 (11.6)
Interobserver	3.9 (8.3)	20.9 (45.0)

Single limit of agreement (LoA) was calculated using average of upper and lower limit. FMA, facial maxillary angle; FNMA, fronto–naso–mental angle; IFA, inferior facial angle; MNMA, maxilla–nasion–mandible angle.

When adding the widest possible random error (95% LoA), as calculated in the intraobserver reproducibility study, to the original cut‐offs for the diagnosis of micrognathia, the percentage of fetuses that potentially met the criteria for diagnosis decreased to 0%, 87%, 49% and 45% for IFA, FNMA, MNMA and FMA, respectively.

## DISCUSSION

The aim of this study was to describe the most reproducible objective ultrasound techniques for the antenatal diagnosis of micrognathia. To the best of our knowledge, this is the first study to compare multiple quantitative methods for the assessment of fetal micrognathia on confirmed cases that were suspected antenatally. The antenatal ultrasound diagnosis of fetal micrognathia can be made subjectively or objectively by assessing the midsagittal fetal facial profile in the second and third trimesters of pregnancy^1^. This is different from postnatal diagnosis, where the mandible shape and ossification are assessed in the coronal view on X‐ray or computed tomography scan. Therefore, commonly used landmarks that define the midsagittal plane of the fetal face are the tip of the nose and the rectangular‐shaped maxilla.

We have shown that the diagnostic accuracy of quantitative methods to identify micrognathia antenatally and their reproducibility are highly variable. This is probably owing to the lack of an objective method for diagnosis^20^. Other ultrasound methods have been suggested for the diagnosis of fetal micrognathia, such as measurement of the fetal mandible compared with the biparietal diameter (BPD) throughout pregnancy^22^. Paladini *et al*.^22^ studied 262 normal fetuses and plotted mandibular diameters against gestational age and BPD as independent variables to build growth charts. They demonstrated a linear relationship between mandibular growth and gestational age or BPD, and that micrognathia affected the growth in the sagittal plane more than that in the coronal plane.

In this study, we decided to select only the methods that applied to the fetal facial profile, as these images are more likely to be obtained routinely during midtrimester fetal anomaly scans as first‐line assessment of the fetal face, and they are recommended by the International Society of Ultrasound in Obstetrics and Gynecology for the routine assessment of midtrimester fetal anatomy^23^. We found that all quantitative methods for the antenatal diagnosis of micrognathia in this study had poor reproducibility, with interobserver 95% LoA being between 10% and 45%. This is probably owing to challenges in caliper placement on landmarks involving soft tissues (nose), lack of definition of bone edges within soft tissues (mandible) and lack of definition of three landmark points to define an angle geometrically. The FNMA appeared to be the most reproducible method, probably because it includes multiple bone structures as landmarks, where the interface between a skeletal structure and the skin can be better defined (forehead). This concept applies to other skeletal structures in fetal biometry; for example, head measurements have been shown to be more reproducible than abdominal measurements^18^, ^24^. In this study, interobserver 95% LoA were shown to be wider than in previous reports. Interobserver 95% LoA in this study *vs* the original studies, when available, were 11.4% *vs* 4% for FNMA^13^, 6.3% *vs* 3.27% for MNMA^14^ and 20.9% *vs* 8% for FMA^14^. A possible explanation is that we used multiple operators and multiple parameters were assessed in the same population. This study more probably reflects real‐life clinical scenarios, where it has been shown that reproducibility of measurements is invariably worse than in the original study.

Limitations of this study include the relatively small number of cases. Despite how rare the condition is, the sample size was adequate to answer the current research question. There was no comparison between accuracy of a subjective *vs* an objective method; the latter has been reported to be more reproducible^25^, ^26^. This was beyond the remit of this study. However, a future appropriate study design would involve comparing subjective and objective quantitative methods in a screening population including normal cases and cases of confirmed micrognathia. The use of experienced operators within a research setting might not reflect real‐life practice. However, this may be seen as a strength, as it was not a source of variability. It is likely that there was an overestimation of angle measurement variability, as a small variation in the absolute values can translate into wide variation in the calculated percentages. However, this is representative of clinical practice. Finally, images of variable quality may have introduced selection bias.

Strengths of the study include selection among antenatally suspected micrognathia cases that were confirmed postnatally and had a known outcome, standardization of caliper placement and comparison of four quantitative methods in the same population.

Some authors have suggested the use of three‐dimensional (3D) ultrasound images to reduce interobserver variability. Rotten *et al*.^12^ studied the fetal mandible *in utero* and obtained 3D images. They reported that it is easier to obtain symmetrical views as they are computer generated and that 3D ultrasound can provide the necessary views more easily. In the present study, we considered only two‐dimensional (2D) images, as this is the recommendation for routine screening ultrasound^23^.

In conclusion, our results confirm that quantitative methods for the antenatal assessment of micrognathia are highly variable in terms of reproducibility and rate of diagnosis. However, if a quantitative method is to be used, the FNMA, obtained in a 2D midsagittal view, should be the preferred measurement to support subjective assessment. The FNMA may be the most appropriate candidate when comparing subjective *vs* objective methods and the evaluation of accuracy in a prospective study. Further investigation into the recognition and characterization of fetal micrognathia *in utero* may lead to more accurate antenatal diagnosis and provide improved parental counseling and neonatal management.

## Supporting information


**Figure S1** Bland–Altman plots of intra‐ and interobserver reproducibility of quantitative measurement of fetal profile angles on ultrasound for antenatal diagnosis of micrognathia. (a) Inferior facial angle (IFA). (b) Fronto–naso–mental angle (FNMA). (c) Maxilla–nasion–mandible angle (MNMA). (d) Facial maxillary angle (FMA). Solid lines are mean and dashed lines are 95% limits of agreement.

**Table S1** Comparison of intra‐ and interobserver reproducibility of quantitative measurements of fetal facial profile angles on ultrasound for antenatal diagnosis of micrognathia.

## Data Availability

Data sharing is not applicable to this article as no new data were created or analyzed in this study.
