# Supplementary material for: Antenatal ultrasound diagnosis of fetal micrognathia: validation and reproducibility of quantitative methods
Source: Ultrasound Obstet Gynecol. 2025 Nov 20;67(1):67–72. doi: 10.1002/uog.70137 (PMC12757816; doi:10.1002/uog.70137)

**Figure S1** Bland–Altman plots of **i**ntra- and interobserver reproducibility of quantitative measurement of fetal profile angles on ultrasound for antenatal diagnosis of micrognathia. (a) Inferior facial angle (IFA). (b) Fronto-naso-mental angle (FNMA). (c) Maxilla-nasion-mandible angle (MNMA). (d) Facial maxillary angle (FMA). Solid lines are mean and dashed lines are 95% limits of agreement.

1. Inferior facial angle (IFA): intra-and interoperator reproducibility.


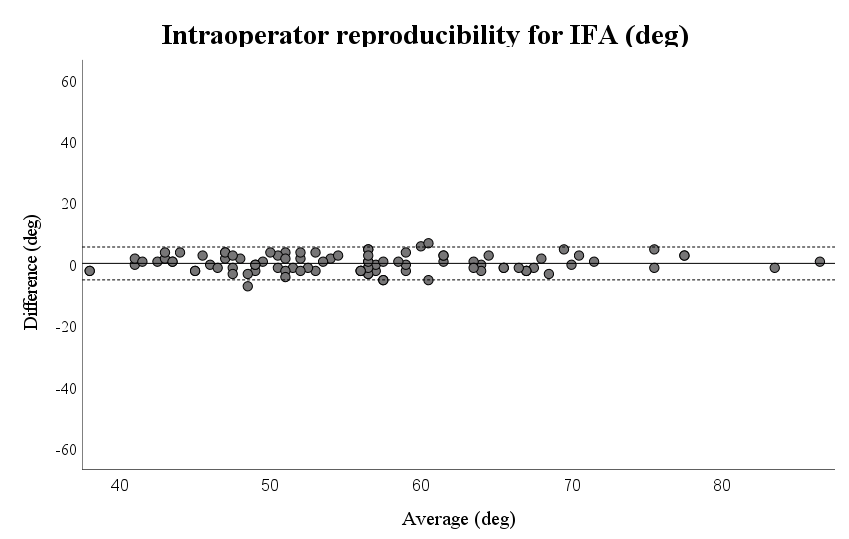

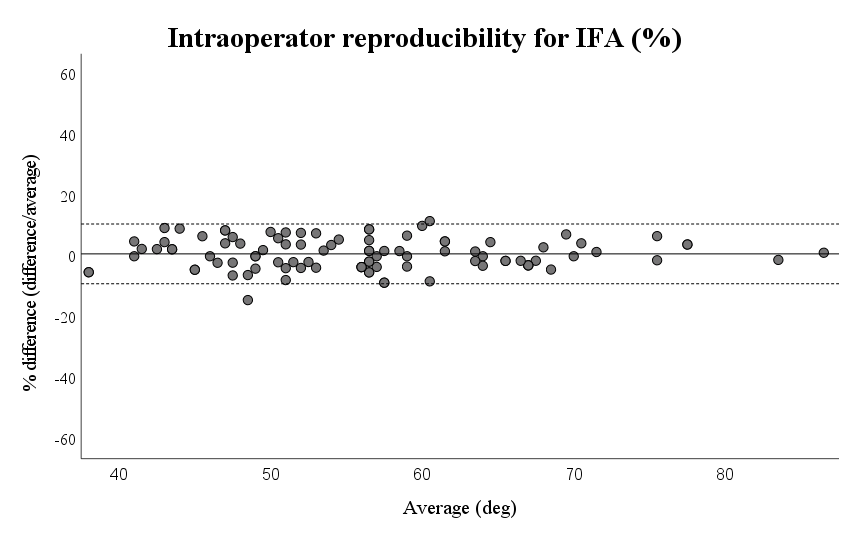

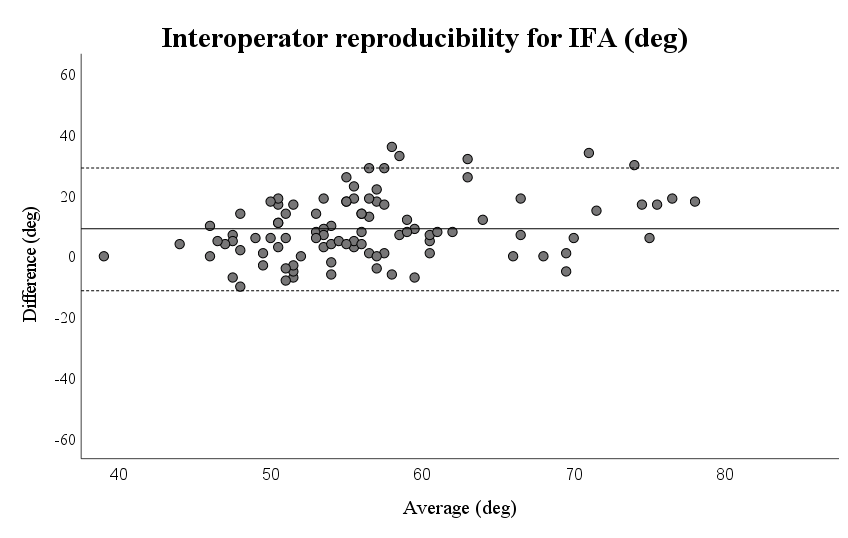

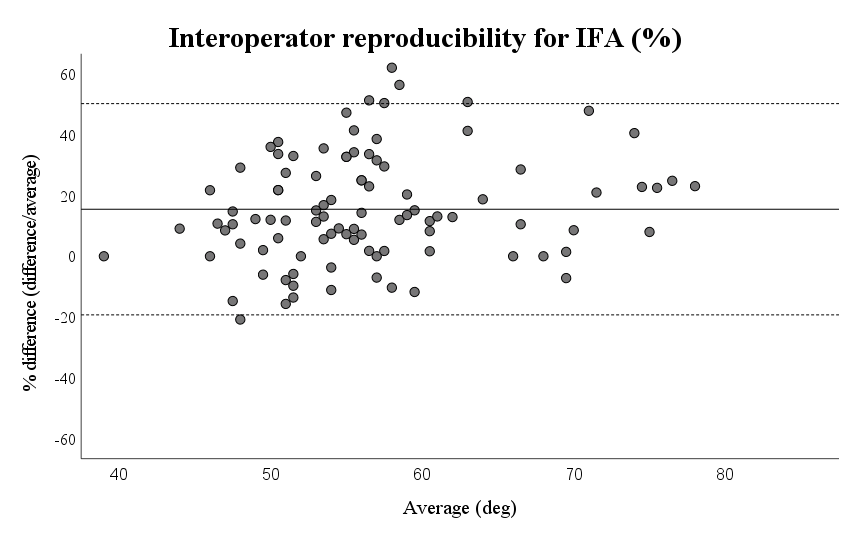


1. Fronto-naso-mental angle (FNMA): intra-and interoperator reproducibility.


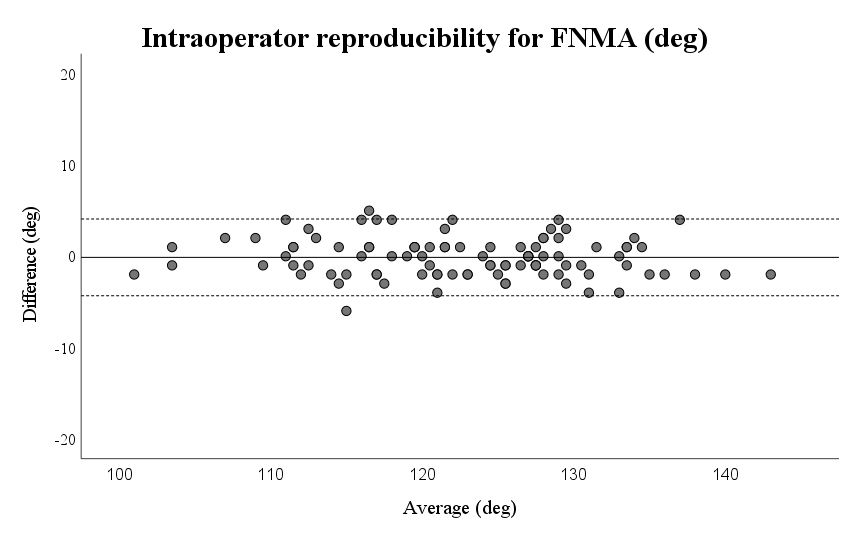

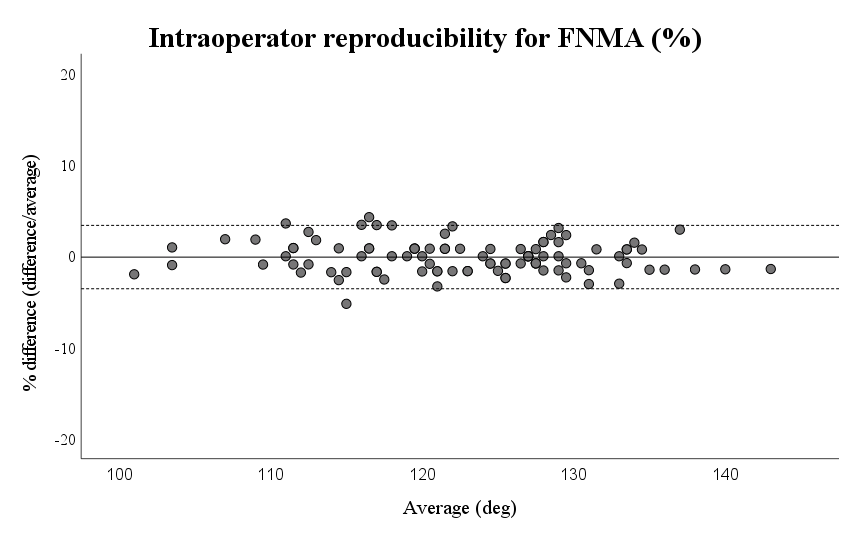


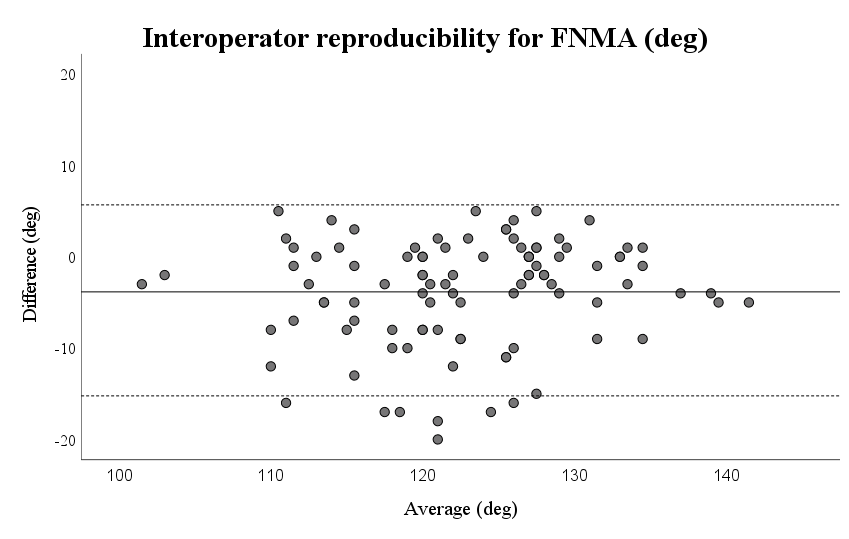

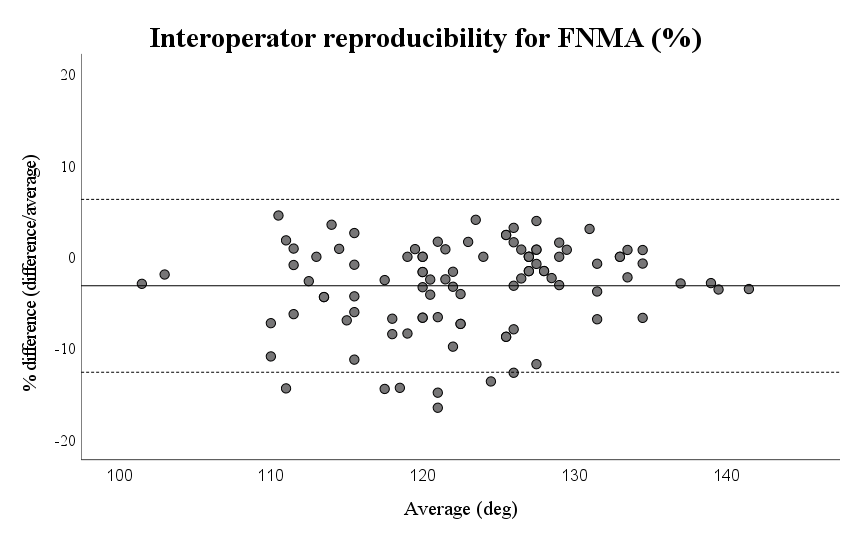


1. Maxilla-nasion-mandible angle (MNMA): intra-and interoperator reproducibility.


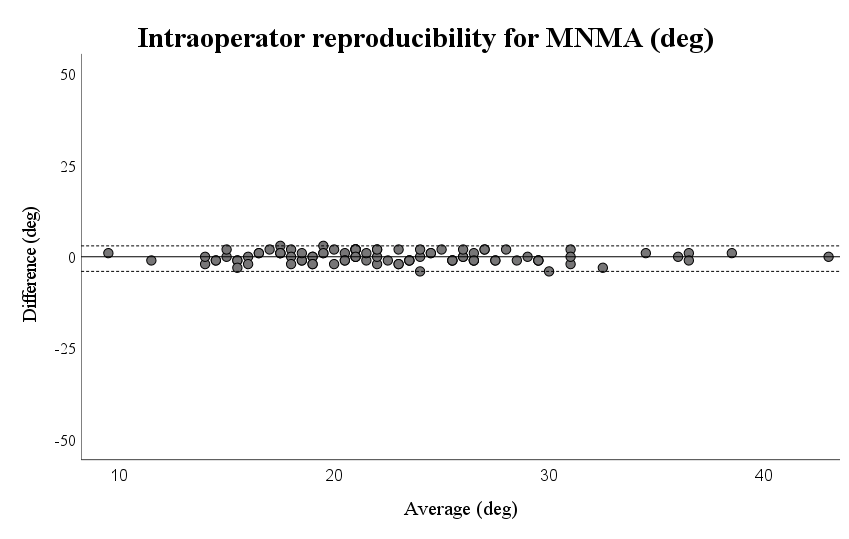

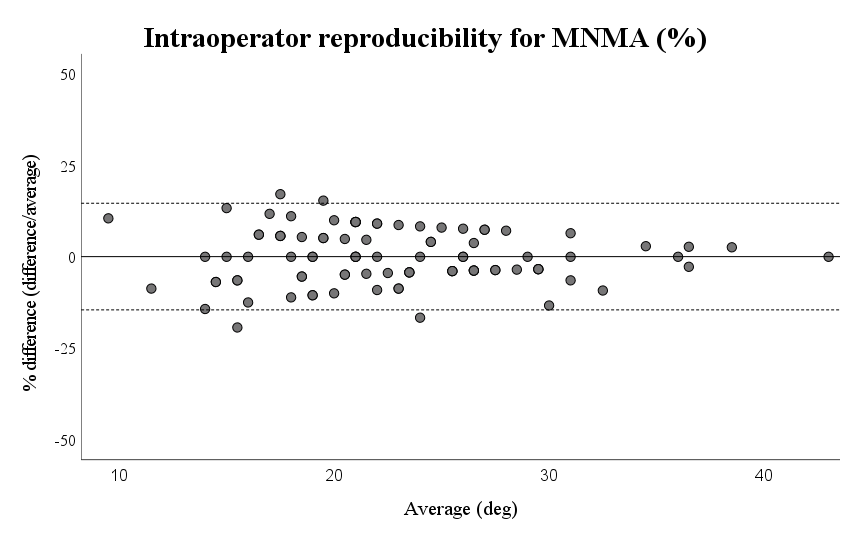


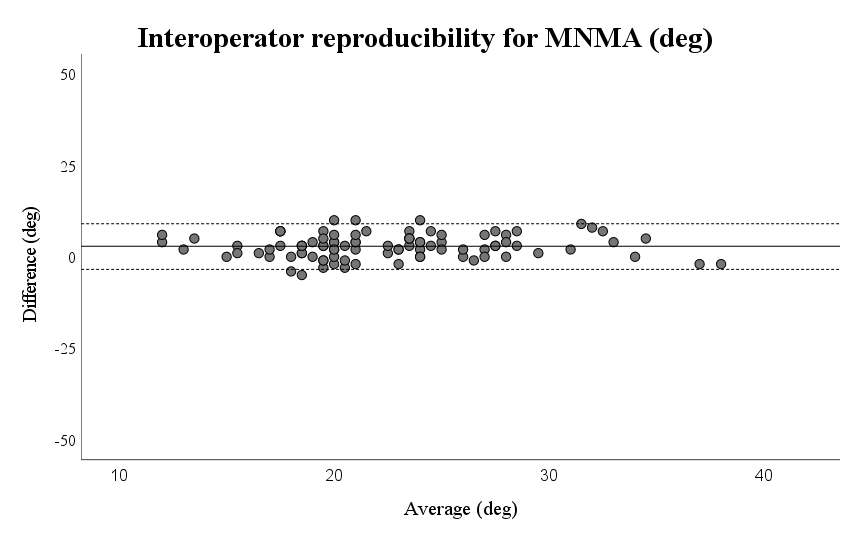

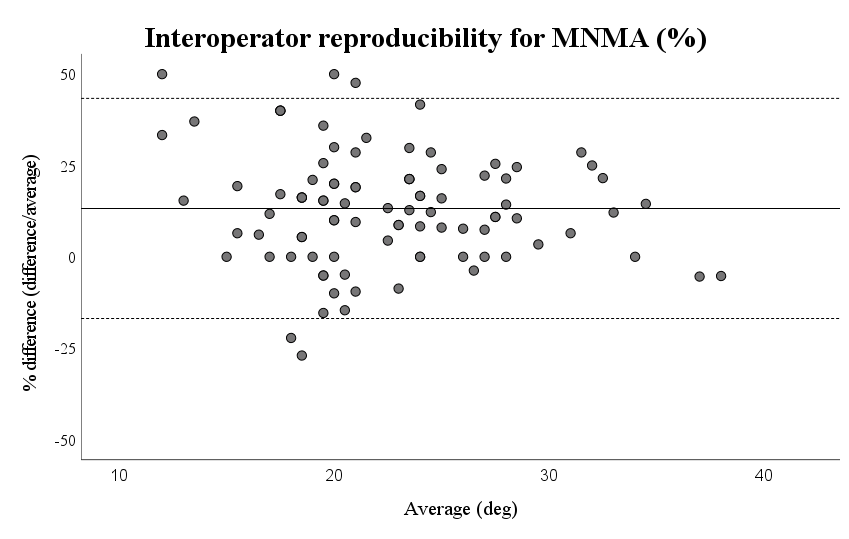


1. Facial maxillary angle (FMA): intra-and interoperator reproducibility


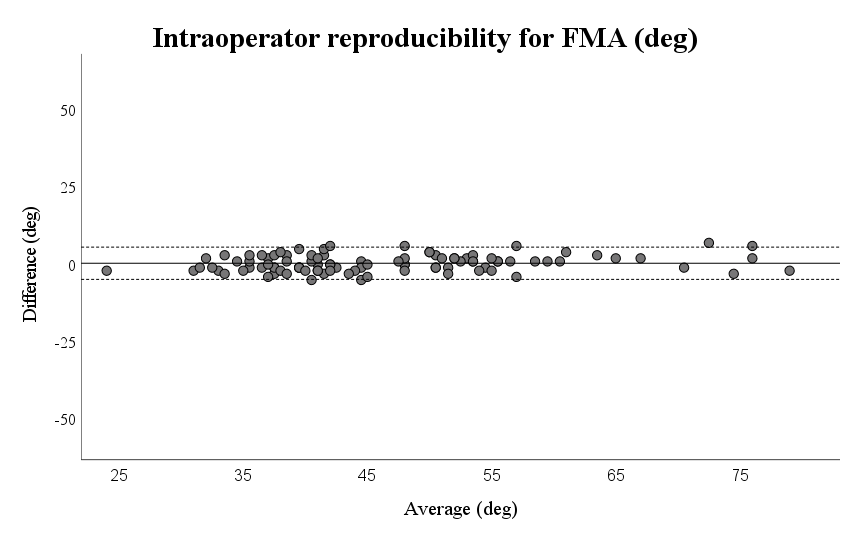

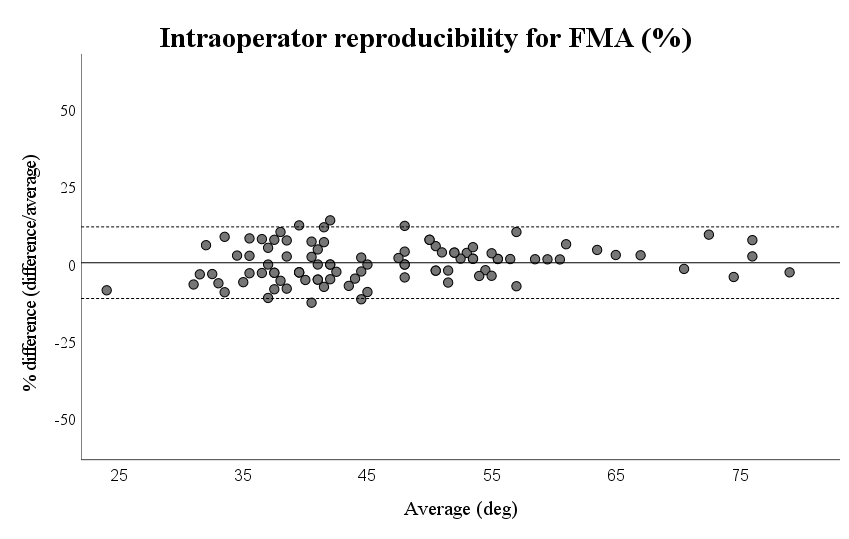


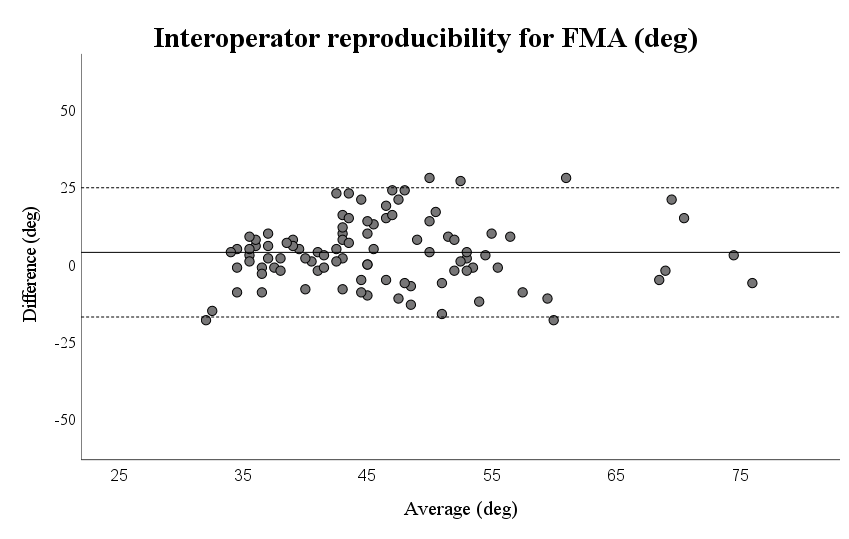

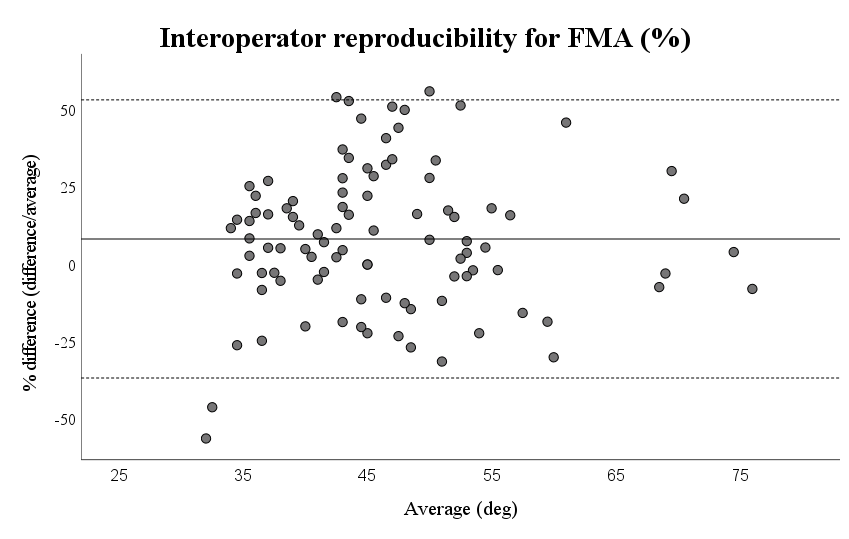

Supplement: Supplementary file 1 — Figure S1 Bland–Altman plots of intra‐ and interobserver reproducibility of quantitative measurement of fetal profile angles on ultrasound for antenatal diagnosis of micrognathia. (a) Inferior facial angle (IFA). (b) Fronto–naso–mental angle (FNMA). (c) Maxilla–nasion–mandible angle (MNMA). (d) Facial maxillary angle (FMA). Solid lines are mean and dashed lines are 95% limits of agreement. [file UOG-67-67-s001.doc]
