# Supplementary material for: Antenatal ultrasound diagnosis of fetal micrognathia: validation and reproducibility of quantitative methods
Source: Ultrasound Obstet Gynecol. 2025 Nov 20;67(1):67–72. doi: 10.1002/uog.70137 (PMC12757816; doi:10.1002/uog.70137)
Supplement: Supplementary file 2 — Table S1 Comparison of intra‐ and interobserver reproducibility of quantitative measurements of fetal facial profile angles on ultrasound for antenatal diagnosis of micrognathia. [file UOG-67-67-s002.docx]

**Table S1** Comparison of intra- and interobserver reproducibility of quantitative measurements of fetal facial profile angles on ultrasound for antenatal diagnosis of micrognathia

|  |  | P-value* |
| --- | --- | --- |
| IFA | Intra- vs interobserver | 0.001 |
| FNMA | Intra- vs interobserver | 0.001 |
| MNMA | Intra- vs interobserver | 0.001 |
| FMA | Intra- vs interobserver | 0.001 |
| IFA vs FNMA | Intraobserver | 0.032 |
|  | Interobserver | 0.001 |
| IFA vs MNMA | Intraobserver | 0.387 |
|  | Interobserver | 0.658 |
| IFA vs FMA | Intraobserver | 0.339 |
|  | Interobserver | 0.003 |
| FNMA vs MNMA | Intraobserver | 0.725 |
|  | Interobserver | 0.001 |
| FNMA vs FMA | Intraobserver | 0.617 |
|  | Interobserver | 0.001 |
| MNMA vs FMA | Intraobserver | 0.996 |
|  | Interobserver | 0.058 |

*Paired sample *t*-test was used for all comparisons. FMA, facial maxillary angle; FNMA, fronto-naso-mental angle; IFA, inferior facial angle; MNMA, maxilla-nasion-mandible angle.
